# Online and Mobile Interventions for Problem Gambling, Alcohol, and Drugs: A Systematic Review

**DOI:** 10.3389/fpsyg.2017.00954

**Published:** 2017-06-09

**Authors:** Isabelle Giroux, Annie Goulet, Jonathan Mercier, Christian Jacques, Stéphane Bouchard

**Affiliations:** ^1^Centre québécois d'excellence pour la prévention et le traitement du jeu, Université LavalQuébec, QC, Canada; ^2^Cyberpsychology Laboratory of UQO, Université de Québec en OutaouaisGatineau, QC, Canada

**Keywords:** psychological intervention, Internet, mobile application, addiction, drug, alcohol, gambling

## Abstract

Online interventions for gambling, alcohol, and illegal drug related problems have been developing at a fast pace over the past decade. Yet, little is known about the content and efficacy of interventions provided entirely online for reducing drug/alcohol use and gambling, or about the characteristics of those who use these interventions. This systematic review aims to describe the characteristics of online interventions, their efficacy, and the profile of their clientele. Documentation was mainly obtained through four scientific databases in psychology, technology, and medical research (PsychINFO, MedLine, Francis, and INSPEC) using three keywords (substances or gambling, intervention, Internet). Of the 4,708 documents initially identified, 18 studies meeting admissibility criteria were retained and analyzed after exclusion of duplicates and non-relevant documents. No study in the review related to problem gambling. The majority of interventions were based upon motivational or cognitive-behavioral theoretical approaches and called upon well-established therapeutic components in the field of addictions. The participants in these studies were generally adults between 30 and 46 years old with a high school education and presenting a high risk or problematic use. More than three quarters of the studies showed a short-term decrease in use that was maintained 6 months later, but only two studies included a 12 months follow-up. Online interventions seem promising and appear to meet the needs of participants who are in the workforce and seeking help for the first time. Long-term efficacy studies should nonetheless be conducted.

## Introduction

Substance use disorder, including alcohol and different classes of drugs, can be defined as a group of cognitive, behavioral, and physiological symptoms indicating that an individual continues using the substance despite significant substance-related problems (American Psychiatric Association, 2013). Gambling behaviors activate reward systems similar to those activated by drugs and produce symptoms comparable to those produced by the substance use disorders (American Psychiatric Association, 2013), and Gambling disorder is the only behavioral addiction with sufficient research to be included in the Substance-related and addictive disorders category of the *Diagnostic and Statistical Manual of Mental Disorders* (5th Edn.; *DSM–5*; American Psychiatric Association, 2013). Systematic reviews of literature report 1 year prevalence rates of 6.6% for alcohol use disorders, 2.4% for other substance use disorders (Somers et al., 2004), and wide variations in past-year problem gambling rates across different countries (0.12–5.8%; Calado and Griffiths, 2016).

Initiating and complying with treatment can be a challenge for clienteles presenting alcohol, drug, or gambling-related problems. In order to overcome the obstacles inherent to psychological interventions and to reduce costs associated with addictions and non-consultation (Sacks et al., 2015), online, and mobile application interventions are developing at a fast pace (Lal and Adair, 2014). What content do they offer to users? Are they effective for reducing drug/alcohol use and gambling? Who is participating in this type of treatment? This systematic review will help to answer these questions.

Even though several psychosocial interventions have shown their efficacy for treating drug, alcohol (Dutra et al., 2008; Magill and Ray, 2009), and gambling disorders (Pallesen et al., 2005; Gooding and Tarrier, 2009), help-seeking rates are low. Only 36% of problem drinkers (Cunningham and Brelin, 2004) and 18% of problem gamblers seek formal assistance (Suurvali et al., 2008). Several obstacles curb help-seeking: need for anonymity or autonomy, shame, and denial (Suurvali et al., 2009; Priester et al., 2016), as well as limited availability (Rockloff and Schofield, 2004) or accessibility of interventions (i.e., scheduling conflicts, transportation difficulties; Clarke, 2007; Priester et al., 2016).

Self-help interventions could help circumvent these obstacles. These interventions are based on self-directed consultation of workbooks which include readings, useful tips and exercises that can be completed at home (Mains and Scogin, 2003; Swan and Hodgins, 2015; Andersson et al., 2016). Based on cognitive-behavioral and motivational approaches (Schmidt and Wykes, 2012; Swan and Hodgins, 2015), self-help interventions may be combined with telephone support provided by a therapist (Mains and Scogin, 2003; Swan and Hodgins, 2015). Several self-help interventions have demonstrated their benefits for treating problem gambling (Labrie et al., 2012; Giroux et al., 2015) and substance use (Carroll et al., 2008; Newman et al., 2011).

Offering self-help material online is a popular alternative in the Internet era. These online interventions first appeared in the 1990s (Childress and Asamen, 1998) and research on the topic multiplied between 2007 and 2010 (Lal and Adair, 2014). According to the findings of two systematic reviews, cognitive-behavioral interventions via computer for various mental health disorders (i.e., depression and anxiety) show an efficacy similar to that of standard face-to-face treatments (Barak et al., 2008) and present a better cost-efficacy ratio (Musiat and Tarrier, 2014). Considering the accessibility of the Internet and the growing popularity of mobile applications, online intervention for substance or gambling disorder could counteract several obstacles related to traditional “offline” services. On-line interventions have the potential to cover large areas at low costs and reach populations that are harder to reach (Barak et al., 2008).

To date, systematic reviews and meta-analyses that targeted the efficacy of online interventions for alcohol, drug, and gambling related problems included several self-help intervention formats (online, telephone, CDRoM, bibliotherapy), delivered alone or in combination, without necessarily differentiating them (for example, Tait and Christensen, 2010; McKellar et al., 2012; Tait et al., 2013; Danielsson et al., 2014; Takano et al., 2015). As such, these studies draw conclusions from a substantial heterogeneity of interventions. Moreover, the inclusion of different types of psychosocial interventions, including preventive interventions, hinders specific observations about interventions targeting the online psychological treatment of addictions. In addition, the participants of these studies have heterogeneous alcohol/drug use and gambling habits; ranging from abstinent, recreational and non-problem users, to those presenting a risk or disorder related to alcohol/drug use or gambling (for example, Tait and Christensen, 2010; Tait et al., 2013). These different participant groups surely have different motivations for signing up for an online intervention program, thus potentially biasing conclusions drawn about the efficacy of these interventions.

Indeed, syntheses on self-help online interventions for addiction do not give a full profile of users participating in online intervention efficacy studies. This information is relevant since these interventions may potentially attract individuals who are not interested in standard interventions (McKellar et al., 2012). For example, Cunningham et al. (2011) showed that at risk alcohol users who completed an online intervention were older, used the Internet more often, and consumed less alcohol during episodes of heavy drinking as compared to those who dropped out of the program. Postel et al. (2011) observed that alcohol users who registered for an online treatment were in greater proportion women, older, more educated, and more likely to be employed and seek treatment for the first time, as compared to those who used a standard treatment.

A literature review pertaining only to online psychological interventions and only to individuals who wish to modify their alcohol, drug, or gambling behavior will help clarify the current state of findings on these interventions. Indeed, a review would shed light on the efficacy of online treatment for drug, alcohol, and gambling problems, and help identify areas of research that need further investigation. Given the diversity of online interventions and technologies developed in recent years, the timing seems appropriate to collect and analyze current available research data. In short, such a review is a step toward better informing public decision-makers, stakeholders and researchers who want to look into new technologies to improve and increase accessibility and adherence to treatments.

The goal of this systematic review is to summarize current knowledge regarding psychological interventions provided entirely online (via computers or mobile applications) for at risk or problem gamblers or users (alcohol, illegal drugs) and that were assessed for efficacy. This review aims to: (a) identify the objectives of the interventions; (b) describe their characteristics (theoretical approach, main components) and modalities (duration, frequency); (c) report the efficacy of interventions in reducing alcohol/drug use and gambling; (d) shed light on participant characteristics; and (e) report the methodological quality of the studies.

## Materials and methods

### Research strategies

Studies relating to online treatment of alcohol, drug (excluding prescribed medication) or gambling related problems were identified via the PsycINFO, MedLine, Francis, and INSPEC databases. Two research strategies were used. First, all databanks were searched with three groups of keywords within the entire text:

(gambl^*^ OR “substance misuse” OR “substance abuse” OR “substance addic^*^” OR “substance dependence” OR “substance related disorder” OR “drug abuse” OR “drug misuse” OR “drug addic^*^” OR “drug dependence” OR “alcohol abuse” OR “alcohol misuse” OR “alcohol addic^*^” OR “alcohol dependence”) AND (online OR app OR apps OR computer^*^ OR smartphone^*^ OR “mobile phone^*^” OR virtual OR “mobile device^*^”) AND (treatment^*^ OR intervention^*^ OR therap^*^ OR “online therap^*^” OR “online intervention^*^”)

Second, PsycINFO database thesaurus and its equivalent in MedLine—the “MESH terms” were used. The group of keywords for the PsycINFO database was:

({Alcohol Abuse} OR {Alcoholism} OR {Drug Abuse} OR {Drug Addiction} OR {Gambling} AND ({Computer Assisted Therapy} OR {Online Therapy})

For the MedLine database, the group of “Mesh terms” was the following:

(“Gambling”[Mesh]) OR “Substance-Related Disorders”[Mesh]) AND (“Therapy, Computer-Assisted”[Mesh]) OR “Drug Therapy, Computer-Assisted”[Mesh])

A search conducted on the http://www.clinicaltrial.org site made it possible to obtain references for unpublished studies and to contact their authors when inclusion criteria were met.

### Inclusion and exclusion criteria

Studies meeting the following criteria were included: (1) written in English or French, (2) published between January 1991 and June 2015 inclusively, (3) involving at least one group whose intervention was entirely online, (4) the intervention targeted the reduction of behaviors or symptoms related to alcohol, drug use, or gambling (5) conducted with users (vs. non-users/abstinents), and (6) used a research design.

Studies were excluded if: (1) the online computer support was used for promoting awareness, prevention (i.e., *personalized feedback*) or strictly for evaluation, (2) the interventions only targeted relapse prevention or consolidation, and (3) they did not include efficacy data.

### Article selection procedure

**Figure 1:**
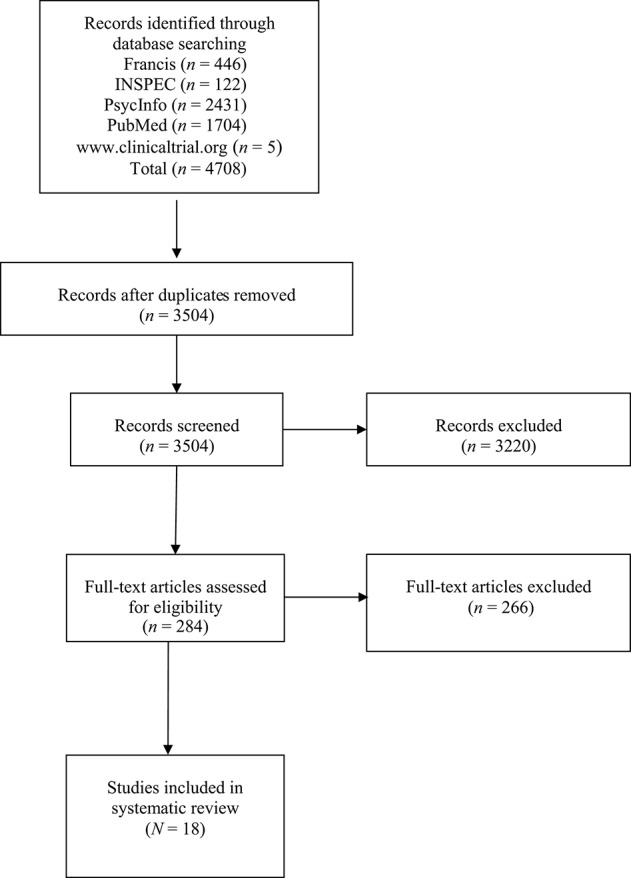
Flow chart displaying the literature search process.

Four thousand, seven hundred and eight studies were initially identified, from which 1,204 duplicates were withdrawn. The titles and abstracts of the remaining 3,504 studies were read and 3,220 non-relevant studies were excluded. Upon the second selection round, 284 studies were fully read to verify inclusion and exclusion criteria. The second selection was the subject of an interrater agreement between two evaluators. The evaluators agreed on criteria for 69.3% of the studies, which was considered unsatisfactory. After clarifying the criteria, the evaluators similarly classified 90.3% of the studies, a satisfactory percentage (Lombard et al., 2002). Disagreements were discussed until consensus was reached. Upon completion of the article selection, 18 studies were retained for analysis. Figure 1 depicts the search process.

### Methodological quality of the studies

The methodological quality of the studies was evaluated using a list of 11 criteria recommended by the Cochrane Back Review Group (Berger and Alperson, 2009). For a study to be judged as possessing good methodological quality, a minimal threshold of six out of the 11 criteria must be met (Berger and Alperson, 2009). The evaluation scale for each criterion is as follows: *Yes* (+), *No* (−) or *I don't know (?)*. Since four criteria were not applicable within the context of this review, only seven of the proposed criteria were retained and rated. The more a study obtains positive ratings, the more it possesses methodological elements of good quality.

### Data analysis

An extraction grid was developed and filled for each study. The information was collected based on eight themes: (1) type of study, (2) methodology, (3) description of participants, (4) intervention objectives, (5) main findings, (7) conclusion, and (8) methodological quality.

The study coordinator and five psychology doctoral students participated in data extraction. An undergraduate research assistant counter-verified the information extracted for the 18 studies.

## Results

### Description of online interventions

To facilitate the reading of the Results section, the 18 studies are referenced using exponents (see Table 1).

**Table 1:** Characteristics of Online Interventions (Objectives a and b) According to Type of Problem (Alcohol and Drugs).

**Studies**	**Name/type of intervention(s)**	**Intervention goal(s)**	**Theoretical model(s)**	**Online availability**	**Intensity (Frequency/Duration/Completion)**	**Format (Techniques/Exercises)**
**PROBLEM: ALCOHOL**						
^1^Blankers et al. (2011)	Self-help Alcohol Online (SAO)	↓ Use	CBT Motivational	Computer Smart phone	Each day (variable)/At any time/–	*Four modules*: Self-observation and self-recording of use, goal setting, personalized feedback, relapse prevention and motivational strategies, discussion forum among participants
	Therapy Alcohol Online (TAO)	↓ Use	CBT Motivational	Computer Smart phone	1 session per day/7 days/40 min per session	*Therapy sessions with a CBT therapist:* Self-recording, goal setting, self-control, management of at-risk situations, urge to drink and emotions, relapse prevention, and consolidation of gains
^2^Braitman (2012)	Alcohol 101 + Booster	↓ Use	–	Computer	Once/–/60 min	*Psychoeducation and interactive virtual campus:* Psychoeducation, normative personalized feedback, strategies relating to attitudes toward alcohol, interpersonal skills. Booster after 2 weeks: feedback
^3^Brendryen et al. (2014)	Balance (self-treatment on alcohol)	↓ Use	Self-regulation CBT	Computer	–/62 sessions over 6 months/3–10 min per session	*62 sessions:* Assessment of use and normative personalized feedback, goal setting, emotional regulation, psychoeducation, relapse prevention, interactive tasks, homework
^4^Brief et al. (2013)	VetChange	↑ Motivation Skills for safe use / cease use	Motivational Self-control CBT	Computer	1 module per week / 8 weeks / 20 min per module	*Eight modules*: Personalized feedback, decisional balance and goal setting with a change plan, at-risk situation management, strategies for managing internal difficulties leading to use, support network development
^5^Carey et al. (2011)	Alcohol 101 plus	↓ Use	–	Computer	Once/–/Minimum 60 min	*Psychoeducational and interactive virtual campus:* Decision-making relating to use, psychoeducation and knowledge test about alcohol
	Alcohol Edu for Sanctions	↓ Use	–	Computer	Two times/–/2 h (1 h/time)	*Five chapters*: Interactive exercises, personalized feedback, quiz questions and risk assessment 1 month later: Reflections about one's personal experience and consolidation of gains
^6^Cunningham (2012)	Check Your Drinking + Alcohol Help Center	↓ Use	Motivational CBT Relapse prevention	Computer	–/Repeated use (−)/–	*Three modules*: Personalized feedback, exercises to initiate change, problem-solve, and consolidate gains, complementary but optional tools
^7^Gajecki et al. (2014)	PartyPlanner App.	↓ Use	Planned behavior	Mobile application	Variable/5–6 weeks/–	*Simulator and estimate of blood alcohol level:* Before and after, comparison of planning to estimate of the real drinking episode
	Check your BAC	↓ Use	Planned behavior	Mobile application	Variable / 5–6 weeks /–	*Blood alcohol level estimate:* Feedback of estimated blood alcohol level in real-time, strategies to maintain use at a not-at-risk level and psychoeducation
^8^Gonzalez and Dulin (2015)	Location-Based Monitoring and Intervention for Alcohol Use Disorders	↓ or cease use	CBT	Mobile application	–/6 weeks/15 min per psychoeducational component, variable for the tools	*Seven modules:* Psychoeducation, personalized feedback, and tools: identification of at-risk situations, of support network and of alternative pleasant activities, craving management, problem-solving and communication skills, exercises
^9^Hester et al. (2013)	SMART Recovery	–↑ motivation and healthy lifestyle –↓addictive behaviors	Motivational CBT	Computer and/or face-to-face	Variable/Variable/Variable	*Online resources comprised of 4 components and meetings with a therapist:* Craving management, thoughts-emotions-behavior links, balanced lifestyle, and relapse prevention
	Overcoming Addictions	Achieve and maintain abstinence	Motivational CBT	Computer	Minimum once per week/Variable/Variable	*Five modules:* Self-recording, exercises, psychoeducation, craving, and relapse management, relapse prevention, cognitive correction, problem-solving, and balanced lifestyle
^10^Matano et al. (2007)	CopingMatters	↓ Use	–	Computer	Variable/max. 90 days/–	*Moderated and interactive Internet:* Personalized feedback (limited or complete), advice, psychoeducation, interactive exercises, self-recording
^11^Postel et al. (2010)	E-therapy program (Alcohol de BAAS)	↓ or cease use ↑ Health indicators	CBT Motivational	Computer/Device with Internet connection	Every day and 1–2 contacts online with a therapist/90 days/–	*Two parts*: Decisional balance, self-recording, questionnaires about use, personalized advice, thought, and behavior modification, problem-solving, consolidation of gains, relapse prevention
^12^Riper et al. (2008)	Drinking Less	↓ Use	CBT Self-control training principles	Computer/Mobile device	–/6 weeks/15 min (stages 1–2) and–(stages 3–4)	*Internet site (4-step program):* psychoeducation, Decisional balance, goal setting, behavior change, consolidation of gains, relapse prevention, exercises and self-recording
^13^Riper et al. (2009)						
^14^Sinadinovic et al. (2014)	CBT-based online extended self-help intervention (Alkoholhjalpen.se)	↓ Use	Motivational CBT	Computer	Variable/Variable/Variable	*Internet site (18 modules):* Identification of at risk situations, decisional balance, relapse prevention, psychoeducation, interactive exercises, questionnaires, and personalized recommendations
^15^Tensil et al. (2013)	Change Your Drinking (original version)	↓ Use	Solution-focused	Computer	Once/10 days/–	Alcohol use journal, objectives, psychoeducation, control strategies, and personalized feedback
	Change Your Drinking (revised version)	↓ Use	Solution-focused CBT Relapse prevention	Computer	–/14 days/–	*Additions*: Identification of at-risk situations, self-control strategies, simple motivational feedback, more elaborate personalized feedback
**PROBLEM: DRUG**						
^16^Rooke et al. (2013)	Reduce Your use: How to Break the Cannabis Habit	↓ Use cease use (cannabis)	CBT	Device with Internet connection	–/6 weeks/–	*Six modules:* Psychoeducation, withdrawal management, identification of dysfunctional thoughts, adjustment strategy and social skills learning, consolidation of gains, relapse prevention, feedback
^17^Schaub et al. (2012)	Snow Control	↓ Use or cease use (cocaine)	CBT Motivational Self-control Relapse prevention	Computer	–/6 weeks/–	*Internet site (program comprised of 8 modules and 4 supplementary modules):* Psychoeducation, decisional balance, identification of at-risk situations, management of urge to use, relapse, free time, saying no skills, and consolidation of gains
^18^Tossman et al. (2011)	Quit the Shit	↓ Use or cease use (cannabis)	Solution-focused	Computer	–/50 days/Steps 1–4: –, 2: 50 min, 3: 50 days	*4-step program:* Initial evaluation through chat with a therapist, goals for ↓ use and management strategies, self-recording, feedback through chat with a therapist, use management strategies

–*, Information absent from the article; ↓, Decrease; ↑, Increase/Improvement; CBT, Cognitive-behavioral theory; FAST, Fast Alcohol Screening Test; max., maximum*.

Eighteen studies were included in the review and these studies evaluated 22 different interventions in total. Fifteen studies^1 to 15^ evaluated 19 interventions for alcohol use. As shown in Table 1, 19 interventions are presented for alcohol use since five studies^1, 5, 7, 9, 15^ compared the efficacy of two interventions and two studies^12, 13^ evaluated the same intervention. The three remaining studies^16, 17, 18^ evaluated an intervention relating to drug use (two for cannabis^16, 18^ and one for cocaine^17^). No study in the review related to problem gambling.

Three of the 22 interventions^7, 8^ are mobile applications for alcohol use, while the others are online interventions (86.4%; ^1 to 6, 9 to 18^).

#### Theoretical models and therapeutic objectives

Thirteen interventions were based, at least in part, upon the cognitive and/or behavioral model (59.1%; ^1, 3, 4, 6, 8, 9, 11, 12, 14, 16, 17^), nine upon a motivational approach^1, 4, 6, 9, 11, 14, 17^, three upon a solution-focused approach^15, 18^, and three upon a relapse prevention model^6, 15, 17^. Self-regulation theories^3, 4, 12, 17^ and planned behavior^7^ underpin certain interventions, whereas for four interventions^2, 5, 10^, the theoretical approaches were not indicated.

Of the 22 interventions, 14 (63.6%; ^1 to 3, 5 to 7, 9, 10, 12, 15^) targeted decreasing use, seven^4, 8, 11, 14, 16 to 18^ offered the choice between decreasing use and abstinence, and only one^9^ targeted abstinence.

#### Techniques and interventions

The majority of interventions (90.9%; ^1 to 6, 8 to 18^) used standard self-help therapeutic material: self-report assessment, self-recordings of use, exercises, readings, and videos. This material aimed, for example, to help the individuals identify at risk situations, determine their goals, modify their thoughts, develop problem solving and emotion management strategies, and to prevent relapse. In addition to this material, four interventions^1, 9, 11, 18^ included online chatting with a clinician, and only one^1^ involved participating in a discussion forum.

Two interventions via mobile applications differed from the other interventions (9.1%; ^7^); the *Check your BAC* and *PartyPlanner App* programs. These interventions enabled users to plan and simulate their alcohol use for a given event and receive an estimate of their blood alcohol levels in real-time.

#### Intensity

Table 1 describes the intensity of the interventions in terms of total duration, frequency of use and completion time. For three interventions (13.6%; ^2, 5, 15^), users' online participation was required only once or twice, whereas the other online interventions lasted between 1 week and 6 months, with suggested utilization of variable frequency (for example, ^6, 7, 9, 10, 14, 16, 17^).

### Profile of the online intervention participants

The sample characteristics for each of the studies are shown in Table 2. A description of the participants is provided for 13 of the 18 studies: For the five other studies, the participants were recruited according to their distinctive characteristics; university students^2, 5, 7^, military personnel returning from combat^4^ and workers from a certain workplace^10^. These studies were excluded from the general description that follows, but are presented in the targeted clientele section.

**Table 2:** Description of the Efficacy Studies and the Sample (Objectives c and d) According to Type of Problem (Alcohol and Drugs).

**Study**	**Principal study objective(s)**	**Experimental conditions (*n)*^a^ and assessment times**	**Sample characteristics (mean age, % women, education, % employed, civil status, % prior treatment, problem severity)^a^**	**Instruments used^b^**	**Main findings**
**PROBLEM: ALCOHOL**					
^1^Blankers et al. (2011)	Compare the efficacy of a therapy provided on the Internet and a self-administered treatment via Internet among problematic drinkers	Internet therapy (IT; *68*) Internet self-administered therapy (IST; *68*) Waiting List (WL; *69*) Initial evaluation (T0) 3 and 6 six months after T0	For IT, IST, and WL respectively: *Age:* 41.9 (10.1); 41. (9.6); 43. (9.3) *% women:* 51; 51; 49 *% post sec. education: 97; 94; 89 % employed:* 85; 82; 78 *Severity: M(SD) on the AUDIT*^c^: 18.8 (4.8); 19.6 (5.6); 20.1 (4.9)	AUDIT TLFB (7 days)	3-month follow-up: significant ↓ on the AUDIT and TLFB among participants from the IT and IST vs. those from WL. 6-month follow-up: IT group improved more than the IST group.
^2^Braitman (2012)	Evaluate the ability of a follow-up session to increase the efficacy of an intervention conducted via computer among university students	Alcohol 101*(181*) Alcohol 101+ (*172*) Control (*39*) Initial evaluation 2-week and 4-week follow-up	*% women*: 65.1 *% full-time students*: 96.7 *% single:* 71.9 *% prior treatment:* 4.6	DDQ	At 2-weeks, no difference between the conditions. At 4-weeks, Alcohol 101+ significantly ↓ number of days of use, quantity of alcohol/occasions and blood alcohol level/day of heavy drinking.
^3^Brendryen et al. (2014)	Compare the efficacy of two versions of the *Balance* intervention (brief/intensive self-treatment) on alcohol use	Intensive self-treatment (*125*) Brief self-treatment (*119*) Initial evaluation 2- and 6-month follow-ups	Intensive self-treatment and brief self-treatment, respectively: *Age:* 39 (14); 37 (13) *% women:* 30; 36 *Severity:* M(SD) on the *FAST*^d^: 6.3 (3.0); 6.2 (2.8)	FAST Daily alcohol use	2-month follow-up: no difference between the groups. 6-month follow-up: *Intensive* group ↓ its use more.
^4^Brief et al. (2013)	Evaluate the efficacy of a web-based intervention for drinking among military personnel returning from combat	Intervention (*404*) Control *(193*) Initial evaluation Post-intervention 3-month follow-up	Intervention and Control, respectively: *% women:* 13.9; 13.3 *% prior treatment (N):* 61 *Severity:* M(SD) on the AUDIT: 17.7 (4.8); 17.6 (4.7)	AUDIT QDS (Qty and frequency of use)	Post-intervention: Intervention group significantly ↓ alcohol use. 3-month follow-up, the intervention group continues to significantly ↓ alcohol use.
^5^Carey et al. (2011)	Evaluate the efficacy of an intervention for alcohol use among students to verify if a brief motivational intervention (BMI) is more effective than that on the Web	Face-to-face BMI (*164*) Alcohol 101+ (*172*) Alcohol Edu (*167*) Wait-list (*174*) Initial evaluation 1-, 6- and 12-month follow-ups	*Age:* 19 (0.71) *% women:* 36 *% university students:* 100	DDQ (drinks / week)	1 month: BMI = better results than for Alcohol 101+ and Edu. The men significantly ↓ their use in the three groups vs. only women in the BMI group. 12-months: maintenance for all groups (except for the women).
^6^Cunningham (2012)	Evaluate the efficacy of a bonified *Alcohol Help Center* (ACH) online intervention vs. a validated brief online intervention *Check your drinking* (CYD)	AHC Group (*83*) CYD Group (*87*) Initial evaluation 6-month follow-up	*Age:* 45.2 (12.2) *% women:* 40.6 *% post-sec. education:* 58.8 *% in a relationship:* 39 *% employed***:** 55 *Severity:* M (SD) on the AUDIT: 22.1 (7.6)	AUDIT AUDIT-C Daily alcohol use	6-month follow-up: significant ↓ on the AUDIT and in daily use for both groups - Significantly greater ↓ in number of drinks / occasion for the AHC group.
^7^Gajecki et al. (2014)	Evaluate the efficacy of two applications among students and to explore differences between the sexes.	Check your BAC (*643*) PartyPlanner app. (*640*) Control (*649)* Initial evaluation 7-week follow-up.	*Age:* 24.7 (4.8) *% women:* 51.7 *Severity:* M (SD) on the AUDIT: 10.7 (3.9)	AUDIT DDQ (qty-frequency/ month) eBAC	7-week follow-up: the BAC group significantly ↑ the frequency of occasions to drink vs the control group. This difference is significant for men only.
^8^Gonzalez and Dulin (2015)	Compare the efficacy of the *Location-based Monitoring and Intervention for Alcohol Use Disorders* application (LBMI-A) to an online motivational intervention *Drinker's Check-up* + bibliotherapy (DCU-BT)	1-LMBI-A (*31*) 2-DCU-BT (*29*) Initial evaluation 6-week follow-up	LMBI and DCU, respectively *Age:* 33.6 (6.5); 34.3 (6.2) *% women:* 46.4^*^; 35 *% post-sec. education:* 42.9; 50 *% in a relationship:* 39.3; 35 *% employed:* 78.6; 65 *Severity:* M (SD) *DSM-5criteria:* 7.1 (2.0)^*^; 5.6 (1.9)	DSM-5 criteria TLFB	6-week follow-up: Only the LMBI-A group reported a significant ↑ in number of days abstinent. For both groups: significant ↓ in number of drinks/week and % of days of heavy drinking, but the effect is greater for the LBMI-A.
^9^Hester et al. (2013)	Evaluate the clinical efficacy of the Overcoming Addiction (OA) and the SMART Recovery (SR) online programs for ↓ drinking and its consequences	1- OA (*19*) 2- OA + SR meeting (*83*) 3- SR meeting (*87*) Initial evaluation 3-month follow-up	*Age;* 44.3 (10.9) *% women:* 60.6 *M (SD) years of education:* 16.1 (2.4) *Severity:* M (SD) on the AUDIT: 24.7 (8.1)	TLFB InDUC	3-month follow-up: All groups show significant ↑ in % of days abstinent, significant ↓ in number of drinks/day and significant ↓ on InDUC score. - ↑ in number of meetings = ↑ number of days abstinence and ↓ in problems.
^10^Matano et al. (2007)	Evaluate the efficacy of an online educational program aiming a ↓ in alcohol use in a work environment for low/moderate risk drinkers	Limited feedback Complete feedback (*N = 173*) Initial evaluation 3-month follow-up	*Age:* 39.9 (11.3) *% women:* 77.9 *% no university education:* 16.1 *% employed:* 100 *% in a relationship:* 46.2	AUDIT Self-reported alcohol use	3-month follow-up: significant ↓ of heavy drinking episodes for those receiving complete feedback, both for *low and moderate risk* individuals.
^11^Postel et al. (2010)	Evaluate the efficacy of an online program involving a therapist for an alcohol use related problem.	e-therapy (*78*) Control (*78*) Initial evaluation 3-month follow-up	*Age:* 45.3 (9.8) *% women:* 53.8 *% post-sec. education:* 57.7 *% employed:* 82.1 *% prior treatment:* e-therapy: 24; control: 4^*^Severity: % Dependence: 81.4, Abuse: 10.3	TLFB (7 days) *Diagnostic Interview* of the DSM-IV-TR (*Substance abuse module)*	3-month follow-up: *e-therapy* group significantly ↓ drinking/week vs. control group. - 68% of *e-therapy* under the threshold for problematic use vs. 15 % of control group (sign. difference).
^12^Riper et al. (2008)	Verify the efficacy of a self-administered online intervention (Drinking Less; DL) for ↓ drinking	DL Group (*130*) Control (psychoeducational handout; *131*) Initial evaluation 6-month follow-up	DL and control, respectively: *Age:* 45.9 (8.9); 46.2 (9.2) *% women:* 49.2; 48.9 *% post-sec. education:* 68.5; 71 *% in relationship:* 57.7; 54.2 *% employed:* 72.3; 73.3 *% prior treatment:* 12 *Severity (% problem):* 100	Self-reported alcohol use (7 days)	6-month follow-up: significantly more participants in DL group are low risk, vs. the control group. DL group significantly ↓ in drinks/week.
^13^Riper et al. (2009)^f^	Verify if the results of a controlled randomized study by Riper et al. (2008) in regards to the DL intervention are generalizable to the general population (DL-pop)	DL (*130*) DL-pop (*378*) Initial evaluation 6-month follow-up	DL-pop alone ^g^: *Age:* 44,3 (10.5) *% women:* 52.6 *% post-se. education:* 54.7 *% in relationship:* 61.4 *% employed:* 82.3 *% prior treatment:* 16.4 *Severity (% problem):* 95.2	AUDIT Self-reported alcohol use (7 days)	Results for the DL-pop group are comparable to those found for the DL group in Riper et al. (2008).
^14^Sinadinovic et al. (2014)	Compare an online CBT intervention to online personalized feedback (eScreen.se) and a control group for alcohol related problems	CBT (*212*) Feedback (*211*) Control (*210*) Initial evaluation 3-, 6-, 12-month follow-ups	CBT, Feedback and Control, respectively: *Age:* 43.8 (11.9); 44 (13.6); 44.1 (12.1) *% women:* 55.7; 55; 54.3 *Severity: Mean score on the AUDIT*: 20.1; 21.5; 20.9	AUDIT AUDIT-C	3-month follow-up: all groups show a significant ↓ on AUDIT and AUDIT-C; scores remain stable at 6- and 12 months.
^15^Tensil et al. (2013)	Evaluate whether the revised version of *Change Your Drinking* (CYD) is more efficacious than the CYD-original	CYD-original (*300*) CYD- revised (*295*) Initial evaluation 6-week and 3-month follow-ups	CYD-o and CYD-r, respectively: *Age:* 29.8 (10.3); 29 (9.4) *% women:* 41; 36.6 *% post-sec.:* 32.3; 37.6 *% prior treatment:* 10.3 *Severity: APS score M (SD)*: 2.4 (1.8); 2.3 (1.7)	APS Self-reported alcohol use (7 days)	3-month follow-up: Both groups show ↓ in number of days of use/week, qty of alcohol, number of episodes of heavy drinking, with no difference between the two interventions.
**PROBLEM: DRUGS**					
^16^Rooke et al. (2013)	Evaluate the efficacy of an online program for ↓ or ceasing cannabis use	Intervention (*119*) Control (*111*) Initial evaluation 6-week and 3-month follow-up	Intervention and control, respectively: *Age:* 31.9 (9.6); 30.2 (9.6) *% women:* 40.3; 36.8 *Severity SDS M (SD):* 14 (3.6); 13.8 (3.6)	SDS GAIN-I TLFB (past month)	6-week follow-up: The Intervention group presents a significant ↓ in days of use, quantity used and symptoms of abuse. Maintenance at 3-month follow-up.
^17^Schaub et al. (2012)	Evaluate the efficacy of an online program to control or cease cocaine use	Intervention (*96*) Control (*100*) Initial evaluation 4-, 6- week and 6-month follow-up	*Age:* 34.2 (8.8) *% women:* 21.9 *% post-sec. education:* 75 *% prior treatment:* 20.4 *Severity SDS M (SD):* 8.0 (3.1)	SDS Cocaine use journal	The number of days of abstinence did not change after the intervention, but the quantity of cocaine used ↓ for both groups.
^18^Tossman et al. (2011)	Verify the efficacy of an online program for ↓ cannabis use.	QTS (*863)* Control (*429*) Initial evaluation 3-month follow-up	*Age:* 24.7 (6.8) *% women:* 29.5 *Education (% completed high-school and college):* 58.7 *Severity (%): DSM-IV - dependence:* 92	Dx criteria of the DSM-IV Self-reported cannabis use	At 3-month follow-up, the QTS significantly ↓ number of days of use.

TAO, Therapy Alcohol Online; SAO, Self-help Alcohol Online; M, mean (or mean score); SD, Standard deviation; Sec., secondary; –, Information missing from the article; AUDIT, Alcohol Use Disorders Identification Test; TLFB, Timeline Follow Back technique: Self-reported measure of standard alcohol consumption within a time frame X preceding evaluation; DDQ, Daily Drinking Questionnaire; Significantly = p < 0.05; vs., versus; ↓, Decrease; ↑, Increase / Improvement; Qty, Quantity; FAST, Fast Alcohol Screening Test; QDS, Quick Drink Screen; eBAC, estimated blood alcohol concentration;

* *Statistically significant difference p < 0.05; DSM-5, Diagnostic and statistical manual of mental disorders, 5^th^ edition; InDUC, Inventory of Drug Use Consequences: APS, Alcohol-related Problem Scale (no threshold). SDS, Severity of Dependance Scale; GAIN-I, Global Appraisal of Individuals Needs-Initials*.

a *Information gathered at the time of the initial evaluation. When available, information for the overall sample is provided. The characteristics for which no information was provided in the study is absent from the grid*.

b *Only instruments used to evaluate the severity of problematic use (alcohol or drugs) and changes in use (quantity-frequency) are reported*.

c *On the AUDIT, a score equal or superior to 8 indicates at-risk (at least) alcohol use*.

d *On the FAST, scores vary between 0 and 16. A score superior to 2 indicates at-risk alcohol use*.

e *This efficacy study pertains to the same intervention as in Dulin et al. (2014)*.

f *This efficacy study pertains to the same intervention as in Riper et al. (2008)*.

g *Only the characteristics of the participants from the DL-RW group are presented. See Riper et al. (2009) for those of the DL-RTC group*.

#### Sex and age

Thirteen studies reported participants' sex. Six of them showed a similar distribution between men and women (46.2%; ^1, 8, 11 to 14^), however, a greater proportion of men was found in six studies (46.2%; ^3, 6, 15 to 18^), of which three evaluated an intervention for drug use^16 to 18^. The mean age of the participants from the 12 studies varied between 30 and 46 years^1, 3, 6, 8, 9, 11 to 17^.

#### Education

Three studies did not provide information about education level^3, 14, 16^. Ten studies provided information on level of education, among which eight reported post-secondary studies for 50%^6, 8, 11, 12, 13, 17, 18^ to 90%^1^ of the participants.

#### Occupational status

Six of the 13 studies (46.2%; ^1, 6, 8, 11, 12, 13^) indicated occupational status and showed that between 55 and 82% of participants were employed.

#### Civil status

Four studies indicated civil status (30.8%; ^6, 8, 12, 13^); the proportion of participants in a relationship varied between 35 and 61%^13^.

#### Prior treatment

Four studies (30.8%; ^11, 12, 13, 17^) reported previous treatment, with percentages varying between 4 and 24% of the sample.

#### Drug/alcohol use problem severity

The majority of the interventions from the 13 studies (92.3%; ^1, 3, 6, 8, 9, 11 to 14, 16 to 18^) were applied to individuals with high-risk use or addiction.

#### Participants through targeted recruitment from a population

##### University students^2, 5, 7^

The participants were young adults, aged on average between 19^5^ and 25 years^7^, studying full-time. Thirteen studies reported the sex of participants. Six reported an even distribution of men and women (46.2 %; ^1, 8, 11 to 14^), however, a majority of men were found in six studies (46.2 %; ^3, 6, 15 to 18^), including the three studies evaluating a drug-related intervention. One study^2^ indicated that 4.6% of students received prior treatment and another^7^ showed at risk alcohol use among participants.

##### Military personnel returning from combat^4^

The participants were men (86%) who had received a prior psychological treatment (61%) and who presented problematic alcohol use.

##### Workers from a targeted workplace^10^

All the participants were employed, the majority were women (78%) and had completed university studies (84%).

### The efficacy of online interventions

Table 2 presents the efficacy of the interventions for bringing changes on at least one substance use indicator (i.e., frequency of use per week or month, amount of use per occasion).

#### Short-term efficacy

Fifteen of the 18 studies (83.3%; ^1 5, 7 to 11, 14 to 18^) evaluated post-intervention changes in substance use. These evaluations took place immediately after the intervention or within a delay varying between 2 weeks and 3 months after initial evaluation. The duration of the interventions being variable within a same study and between studies, the evaluation that takes place 3 months after initial evaluation could be considered close to the end of the intervention.

Eleven studies reported a significant decrease on at least one substance use indicator for the group receiving the online intervention, seven as compared to a control group without intervention^1, 2, 4, 5, 11, 16, 18^, two as a group receiving another type of intervention^8, 10^, and two without a control group^9, 15^. However, four studies obtained mitigated results: a significant decrease in severity of alcohol related problems among all participants, including those in the group that received no intervention^14^, very limited efficacy^17^, or absence of efficacy^3^ as compared to a group without intervention, and even a lack of efficacy with an increase in substance use episodes among men^7^.

#### Medium and long-term efficacy

Eight of the 18 studies (44.4%; ^1, 3, 5, 6, 12, 13, 14, 17^) included a 6-month post initial evaluation follow-up. Overall, these studies showed the maintenance of improvements observed shortly after intervention. Only two studies^5, 14^ evaluated the maintenance of gains 12 months after the initial evaluation, of which one^5^ showed that the gains were maintained exclusively for women, whereas the other^14^ study showed maintenance of improvements for all groups, including the group who did not received intervention.

### Methodological quality of the studies

Table 3 presents the evaluation of the studies based on the seven criteria in decreasing order of methodological quality score. Nine studies met six criteria (50%; ^4, 6, 7, 9, 14 to 18^), two satisfied five criteria^3, 12^, and six met four criteria^1, 2, 8, 10, 11^. One study met only two criteria^13^. Half of the 18 studies^2, 3, 5, 6, 8, 10, 12, 13, 16^ presented insufficient information to determine if they met at least one criterion.

**Table 3:** Methodological Quality of the Studies According to Seven of the Eleven Criteria of the *Cochrane Back Group Criteria List* (objective e), According to Type of Problem (Alcohol and Drugs).

**Study**	**Criterion**^a^							
	**A**	**B**	**C**	**H**	**I**	**J**	**K**	**Total**
**PROBLEM: ALCOHOL**								
^4^Brief et al. (2013)	+	+	+	+	−	+	+	6
^6^Cunningham (2012)	+	+	+	?	+	+	+	6
^7^Gajecki et al. (2014)	+	+	+	+	−	+	+	6
^9^Hester et al. (2013)	+	+	+	−	+	+	+	6
^14^Sinadinovic et al. (2014)	+	+	+	+	−	+	+	6
^15^Tensil et al. (2013)	+	+	+	+	−	+	+	6
^3^Brendryen et al. (2014)	+	+	?	+	−	+	+	5
^12^Riper et al. (2008)	+	?	+	+	−	+	+	5
^1^Blankers et al. (2011)	+	+	+	−	−	−	+	4
^2^Braitman (2012)	+	+	?	+	−	+	?	4
^5^Carey et al. (2011)	?	?	+	+	+	+	?	4
^8^Gonzalez and Dulin (2015)	−	−	+	+	+	+	?	4
^10^Matano et al. (2007)	+	?	+	+	+	−	−	4
^11^Postel et al. (2010)	+	+	+	+	−	−	+	4
^13^Riper et al. (2009)	−	−	?	+	−	−	+	2
**PROBLEM: DRUGS**								
^16^Rooke et al. (2013)	+	+	+	+	+	?	+	6
^17^Schaub et al. (2012)	+	+	+	+	−	+	+	6
^18^Tossman et al. (2011)	+	+	+	+	−	+	+	6

*+, Yes (criterion met); −, No (criterion not met); ?, Don't know (no indicator in the text making it possible to determine if the criterion is met)*.

Criterion A: Was the method of randomization adequate?

Criterion B: Was the treatment allocation concealed?

Criterion C: Were the groups similar at baseline regarding the most important prognostic indicators?

Criterion H: Was the compliance acceptable in all groups?

Criterion I: Was the dropout rate described and acceptable?

Criterion J: Was the timing of the outcome assessment in all groups similar?

Criterion K: Was the intent-to-treat analysis performed?

a *The criteria D (Was the patient blinded to the intervention?), E (Was the care provider blinded to the intervention?), F (Was the outcome assessor blinded to the intervention?) and G (Were cointerventions avoided or similar?) were not evaluated because of difficulties applying them to the studies reviewed*.

## Discussion

The goal of this systematic review is to portray psychological interventions that are entirely online for people with alcohol, drug, and gambling related problems.

Like the systematic review conducted by Danielsson et al. (2014), this review shows that studies evaluating the efficacy of online interventions for alcohol related problems are more numerous than those for illegal drugs and gambling. Moreover, no efficacy study on a completely online intervention for problem gambling was retained for this study. With its systematic approach, this review highlights the glaring lack of research on the effectiveness of online or mobile applications to help individuals with gambling problems. Online interventions for gamblers were identified, but they were excluded for three main reasons. First, they targeted gambling prevention (personalized feedback) or addressed a student clientele without a gambling problem (for example, Hopper, 2008; Lostutter, 2009). Personalized feedback interventions are closer to secondary prevention than pure psychological intervention to reduce or eliminate an undesirable behavior (Hopper, 2008; Palfai et al., 2014). For the most part, these interventions do not target a specific clientele, as they include non-consumers with high-risk consumers (for example, Cunningham et al., 2006; Doumas and Andersen, 2009; Bewick et al., 2010; Labrie et al., 2013), with the goal to keep their consumption within recommended limits. Nevertheless, these personalized feedback interventions represent an innovative way of raising awareness about the participants' own consumption and may in some cases lead to changes in behavior. Second, these interventions were not offered completely online, as some studies included a telephone contact with a clinician (for example, Carlbring and Smit, 2008). Third, they did not present data about efficacy, like the preliminary study of satisfaction with an entirely online intervention for excessive gambling (see Zermatten et al., 2010). It would be important to prioritize to research into the use of new technologies in the treatment of problem gamblers, considering that standard “offline” self-help treatment programs are effective in reducing negative consequences of excessive gambling behavior (Hodgins et al., 2001, 2009; Giroux et al., 2015), but may not be easily accessible to gamblers. It thus appears necessary to empirically evaluate the efficacy of online interventions dedicated to problem gamblers.

The large majority of online interventions for alcohol or drugs were delivered on a web platform, with the exception of three mobile applications. Two of these applications (see Gajecki et al., 2014) differ from the other interventions in regards to their theoretical approach, that of planned behavior (Ajzen, 1991), and their content, which consisted of a behavioral tool to manage alcohol use and estimate blood alcohol levels in real time. Such interventions show that technological advances can potentially diversify intervention tools for users. Yet, as the review suggests, these interventions may lead to undesirable effects such as an increase in substance use occasions. These findings support the need to rigorously evaluate their efficacy and to require the same quality standards as for face-to- face treatments.

In regards to the therapeutic objectives targeted by online interventions, the majority of them offer a freedom of choice to the users as to goals of decreasing use or abstinence. They also offer users flexibility when targeting goals for decreased alcohol/drug use. Imposing abstinence may discourage many users from seeking assistance (Ursúa, 2008) and favor treatment withdrawal, even before treatment begins (Andrewartha and Dowling, 2006). The flexibility offered by online interventions may favor treatment adherence and even lead to a change of therapeutic objective toward abstinence, as observed in a large portion of the sample of gamblers receiving face-to-face treatment in the study conducted by Ladouceur et al. (2009).

The majority of online interventions are based on cognitive-behavioral or motivational approaches; approaches underpinning standard and self-administered psychological treatments for gambling and alcohol/drug related problems (Dutra et al., 2008; Cowlishaw et al., 2012; Martin and Rehm, 2012). These two approaches are recognized as efficient for addiction treatment and their structure, as well as the techniques used, appear to be easily adaptable to an online format (Gainsbury and Blaszczynski, 2011). This review suggests that for several interventions, the full potential of a web platform does not appear to be exploited. Indeed, with the exception of only a few interventions, the techniques and tools used appear to be mostly replicas of what is already offered offline. Only 18.2% of the interventions used an online chat room, while 4.5% offered an online support forum; these tools are very easy to set up, but the content needs to be supervised by a professional. As for mobile application interventions, they tend to exploit the originality of new technologies but may possibly lead to undesirable effects among certain participants; for example, an increase in use in order to use the mobile application more frequently, to see how much their blood alcohol level could rise. Rethinking the way these apps are engineered could help with this novelty effect.

Contrary to standard cognitive-behavioral treatments that give priority to a clear therapeutic framework (Andersson et al., 2016), this review shows that the suggested utilization of the online interventions for participants is variable, or even provided without any defined indicators. The flexibility of the therapeutic framework is typical of self-administered interventions, although those offered offline generally suggest a certain treatment intensity (Simoneau et al., 2004; for example, Carroll et al., 2008; Ladouceur et al., 2015). The malleable and little demanding framework of online interventions may counterbalance obstacles to entering treatment (Priester et al., 2016), but is susceptible to give rise to variable commitment and utilization.

This review made it possible to extract a profile of participants of online interventions for drug and alcohol use outside of targeted recruitment. Overall, the participants are mostly adults between 30 and 46 years old who are educated and employed. Although less than a quarter of the clientele reported having previously consulted for drug or alcohol related problems, the majority was identified as presenting problematic drug/alcohol use.

This profile appears similar to that drawn by Postel et al. (2011). The *online* format attracts adults between 30 and 46 years old, which indicates that this clientele may be more receptive to technological advances because of their active and regular Internet use (Ducharme, 2015). Users who utilize online interventions generally appear to be educated. This is consistent with the proposed type of interventions, which require reading, writing, and computer skills (Farrer et al., 2014). The high proportions of workers may also indicate that the flexibility offered by online interventions fits well within a busy life schedule. Studies show that a higher level of education (Spek et al., 2008) and being employed (de Graaf et al., 2010) are predictors of success on online cognitive-behavioral interventions for depression. The same may apply to interventions for drug and alcohol users, but further studies would be needed to verify this hypothesis. Finally, the fact that few participants had previously consulted suggests that online interventions are attractive for first time consultations; they may demystify treatment and act as a stepping stone toward other more intensive intervention modalities (Bower and Gilbody, 2005).

The online interventions generally seem to be efficacious for reducing certain drug and alcohol use behaviors on a short-term basis. The majority of the studies report positive results on at least one substance use indicator following intervention, and some show a medium term maintenance of gains. These results appear to be coherent with other studies evaluating the efficacy of offline self-help treatments (Hodgins et al., 2009; Giroux et al., 2015). However, few studies in the review evaluate long-term changes and those that do, report little conclusive findings. It thus remains difficult to draw conclusions about the long-term benefits of online interventions for problematic use of drugs/alcohol. These results point to the relevance of including longer-term follow-ups to verify the maintenance of gains over time.

This review presents some limitations. One of them relates to the difficulty of the review to meet the final research objective, that of presenting the methodological quality of the studies. Indeed, even though this grid used Cochrane recommended criteria, the evaluation grid used required the withdrawal of nearly a third of the tool's criteria because of the difficulty applying them to the types of studies identified. Another limitation relates to the subjective portrait offered by the review regarding the efficacy of the online interventions since the review's inclusion criteria, as well as the study objectives, did not make it possible to conduct a meta-analysis.

The review does however possess strengths, notably the scientific rigor employed during the study selection and database extraction processes. A second strength lays in the selection of interventions included in the review; those available entirely online. By differing from the review of Danielsson et al. (2014) that included different intervention modalities, alone or in combination, the present review draws a more homogenous portrait. However, the studies evaluating online interventions for problem gamblers were excluded and the conclusions offered cannot be generalized to this population. To draw a portrait of the users recruited from the general population represents another strength of the review, in that it provides support to empirical studies that have already conducted this exercise and makes it possible to identify the clientele that may be attracted to these online interventions.

In conclusion, this review shows that, in general, psychological interventions offered completely online for alcohol and drugs do not reinvent the underpinnings of self-administered interventions in regards to both the theoretical approach and their content. The online format represents an alternative way to offer these interventions, which could increase accessibility and attract a clientele who would not consult otherwise. These interventions appear promising and have short-term benefits among their users. However, further research is essential. Firstly, it is primordial to evaluate the efficacy of these interventions while including long-term follow-up measures. Secondly, the interventions offered through mobile applications appear to represent a challenge; they are based on less conventional approaches in regards to addiction and show mitigated results. This type of intervention should be further examined in order to ensure their safety. As such, other rigorous scientific studies are needed to be conducted before integrating them into a treatment program. Finally, development and evaluation of interventions that are entirely online for problem gambling are necessary steps to the diversification of intervention tools for this clientele.

## Author contributions

Manuscript was written by IG, AG, JM, CJ, and SB. JM and SB helped with the study design, methodological issues and manuscript revision. Data were collected by AG and JM. The study and the manuscript redaction were supervised by IG. All authors made substantial contributions to the conception or design of the work; or the acquisition, analysis, or interpretation of data for the work; All authors worked on drafting the work or revising it critically for important intellectual content; and all authors approved the version to be published and agreed to be accountable for all aspects of the work in ensuring that questions related to the accuracy or integrity of any part of the work are appropriately investigated and resolved.

### Conflict of interest statement

The authors declare that the research was conducted in the absence of any commercial or financial relationships that could be construed as a potential conflict of interest.
